# Non-invasive ^11^C-Imaging Revealed the Spatiotemporal Variability in the Translocation of Photosynthates Into Strawberry Fruits in Response to Increasing Daylight Integrals at Leaf Surface

**DOI:** 10.3389/fpls.2021.688887

**Published:** 2021-07-14

**Authors:** Yuta Miyoshi, Kota Hidaka, Yong-Gen Yin, Nobuo Suzui, Keisuke Kurita, Naoki Kawachi

**Affiliations:** ^1^Takasaki Advanced Radiation Research Institute, National Institutes for Quantum and Radiological Science and Technology (QST), Takasaki, Japan; ^2^Kyushu Okinawa Agricultural Research Center, National Agriculture and Food Research Organization (NARO), Kurume, Japan; ^3^Materials Sciences Research Center, Japan Atomic Energy Agency, Tokai, Japan

**Keywords:** carbon-11, light period, positron-emitting tracer imaging system, strawberry (*Fragaria* × *ananassa* Duch), photosynthate translocation

## Abstract

The efficiency of photosynthate translocation from leaves to fruits directly affects dry matter partitioning. Therefore, controlling photosynthate translocation dynamics is critical for high-yield and high-quality fruit production. Accordingly, photosynthate translocation changes must be characterized using data obtained at a higher spatiotemporal resolution than those provided by conventional methods. In this study, ^11^C-photosynthate translocation into strawberry (*Fragaria* × *ananassa* Duch.) fruits in individual plants was visualized non-invasively and repeatedly using a positron emission tracer imaging system (PETIS) to assess the spatiotemporal variability in the translocation dynamics in response to increasing daylight integrals (i.e., 0.5-, 4.5-, and 9-h exposures to 400 μmol m^–2^ s^–1^ at the leaf surface). Serial images of photosynthate translocation into strawberry fruits obtained from the PETIS confirmed that ^11^C-photosynthates were translocated heterogeneously into each fruit on the same inflorescence. The amount of translocated ^11^C-photosynthates and the translocation rate into each fruit significantly increased as the integrated light intensity at the leaf surface increased. An analysis of the pedicel of each fruit also confirmed that the photosynthate translocation rate increased. The cumulated photosynthesis in leaves increased almost linearly during the light period, suggesting that an increase in the amount of photosynthates in leaves promotes the translocation of photosynthates from leaves, resulting in an increase in the photosynthate translocation rate in pedicels and enhanced photosynthate accumulation in fruits. Additionally, the distribution pattern of photosynthate translocated to fruits did not change during the light period, nor did the order of the sink activity (^11^C radioactivity/fruit dry weight), which is the driving force for the prioritization of the ^11^C-partitioning between competing organs, among fruits. Thus, this is the first study to use ^11^C-radioisotopes to clarify the spatiotemporal variability in photosynthate translocation from source leaves to individual sink fruits *in vivo* in response to increasing daylight integrals at a high spatiotemporal resolution.

## Introduction

Photosynthate translocation from source organs (e.g., matured leaves) to sink organs (e.g., fruits, new shoots, and roots) is an important physiological process that directly affects dry matter accumulation in fruits and promotes the growth of sink tissues, thereby significantly influencing the crop yield and quality (Troughton and Currie, 1977; Braun et al., 2014; Hidaka et al., 2019). The photosynthate translocation to sink organ is generally regulated by the sink strength (Moorby, 1968; Gifford and Evans, 1981; Marcelis, 1996), which is determined by the sink size and sink activity (Warren, 1972). Sink activity reflects the physiological responses in photosynthate translocation (Ho, 1996), and it has been suggested that sink activity rather than sink size is a major determinant of sink strength in strawberry plants (Hidaka et al., 2019). The photosynthate translocation to sink organs is greatly affected by the environmental factors such as light intensity, air temperature, and CO_2_ concentration. To ensure highly profitable and stable fruit production, the translocation induced by environmental conditions must be clarified. The resulting information will be useful for determining the ideal cultivation conditions for optimizing photosynthate translocation into fruits.

Several studies have analyzed photosynthate translocation in response to environmental stimuli. For example, using a ^14^C tracer method, Yoshioka (1986) and Pickard and Minchin (1990) analyzed tomato and *Phaseolus vulgaris*, respectively, and clarified the effects of temperature variations in leaf petioles on the photosynthate translocation rate. Troughton and Currie (1977) and Lanoue et al. (2018) used ^11^C and ^14^C tracers to conduct studies on maize and tomato leaves, respectively, to evaluate the effects of light intensity and light quality on photosynthate translocation. Regarding strawberry plants, the effects of fruit developmental stages, leaf positions, and environmental conditions (light intensity and air temperature) on photosynthate translocation have been analyzed using EDTA to examine the phloem exudates from fruit pedicels as well as ^14^C and ^13^C tracers (Forney and Breen, 1985; Nishizawa and Hori, 1986, 1988; Kumakura and Shishido, 1994; Nishizawa et al., 1998; Hidaka et al., 2014). However, most of the methods used in previous studies to analyze photosynthate translocation dynamics require the destruction and extraction of plant tissues. Moreover, the generated data are fragmented as they are restricted to specific periods. To thoroughly elucidate photosynthate translocation, imaging techniques with a higher spatiotemporal resolution than that provided by conventional techniques are required. Therefore, in this study, we used the ^11^C tracer and the positron-emitting tracer imaging system (PETIS). The ^11^C is one of the short-lived RI tracers that emit positrons, with the half-life of 20.39 min. A major advantage of such tracers is that *in vivo* measurement is possible, giving detailed time series of tracer data in many locations (Minchin and Thorpe, 2003). The PETIS also facilitates the non-invasive, real-time imaging of plant physiological activities. It produces movie-like serial, two-dimensional images of long-distance translocation in plants. Because the half-life of ^11^C is about 20.39 min, photosynthate translocation dynamics in an individual plant can be visualized repeatedly under various environmental conditions, enabling a highly accurate analysis of translocation changes. We previously completed PETIS-based investigations on the spatiotemporal distribution of photosynthates in leguminous plants (Matsuhashi et al., 2006; Kawachi et al., 2011; Yin et al., 2020), *Cannabis sativa* (Kawachi et al., 2006), eggplant (Kikuchi et al., 2008), tomato (Yamazaki et al., 2015; Tsukamoto et al., 2020), and strawberry (Hidaka et al., 2019). Various other studies have been conducted on the elucidation of physiological functions using ^11^C tracers. Thorpe et al. (1998) analyzed the changes in carbon partitioning into soybean rhizoids when root systems were treated with nitric acid. Ferrieri et al. (2005) analyzed the dynamics of ^11^C-isoprene emissions from leaves of poplar seedlings. Babst et al. (2005) revealed that an increase in jasmonic acid, a plant hormonal signal of herbivore attack, leads to more rapid ^11^C-photosynthate export from leaves and greater ^11^C-photosynthate partitioning into stems and roots in Populus species. Mincke et al. (2020) analyzed the internal movement dynamics of ^11^CO_2_ through the xylem inside branches of *Populus tremula* L. In addition, research on detectors for imaging ^11^C continues, and Kiser et al. (2008) have reviewed the versatile imager for positron-emitting radiotracers, a system that enables 2D imaging at a greater resolution than conventional detectors.

The aim of this study was to clarify the spatiotemporal variability in the translocation of photosynthates into strawberry (*Fragaria* × *ananassa* Duch.) fruits in response to the increasing exposure of leaf surfaces to daylight integrals (i.e., light periods). Strawberry is an important horticultural crop grown worldwide. Strawberry fruits provide consumers with a variety of sensory experiences and health benefits because of their pleasant aroma, sweet and sour tastes, and antioxidative properties (Giampieri et al., 2012; Nile and Park, 2014; Afrin et al., 2016; Battino et al., 2016; Warner et al., 2021). Because of their desirable taste and health benefits, large quantities of strawberry fruits are consumed fresh and in processed foods worldwide. The total global production of strawberries has increased over the past two decades, with the yield exceeding 8.8 million tons in 2019 (FAO, 2019). The aroma and taste of strawberry fruits (i.e., quality indicators) are determined by the ratios of the principal soluble components, namely, sugars and organic acids (Perez et al., 1992; Montero et al., 1996; Kallio et al., 2000; Moing et al., 2001). More specifically, sucrose, glucose, and fructose, which are the most abundant soluble solids in strawberry fruits, determine fruit sweetness (Perez et al., 1997; Todeschini et al., 2018). Because the fruit sugar content is greatly affected by the ambient environment, strawberry plants are increasingly being cultivated under controlled environmental conditions (e.g., light intensity, air temperature, and CO_2_ concentration) (Neri et al., 2012; Hidaka et al., 2013, 2016; Miyoshi et al., 2017; Samtani et al., 2019; Yoneda et al., 2020). During this protected cultivation, environmental conditions are modulated to promote leaf photosynthesis, which positively affects strawberry fruit yield and quality. However, to further increase the fruit yield and quality, the effects of the environment on the translocation of photosynthetic products into the fruits must be characterized. The examined photosynthate translocation dynamics may be relevant for developing an environmental control system useful for the protected cultivation of horticultural plants. Earlier research on diurnal variations in photosynthate translocation dynamics focused on the differences between the light and dark periods. In this study, we hypothesized that photosynthate translocation may vary over time, even during the light period. We tested this hypothesis using a PETIS and a ^11^C tracer to determine the spatiotemporal changes to photosynthate translocation from the source leaves to individual sink fruits after the source leaves were exposed to varying daylight conditions.

## Materials and Methods

### Plant Material and Growth Conditions

**FIGURE 1:**
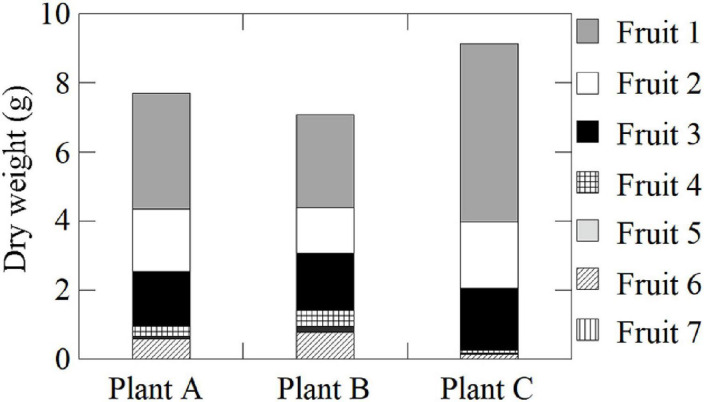
Dry weights of fruits on the same inflorescences of Plants A–C.

**FIGURE 2:**
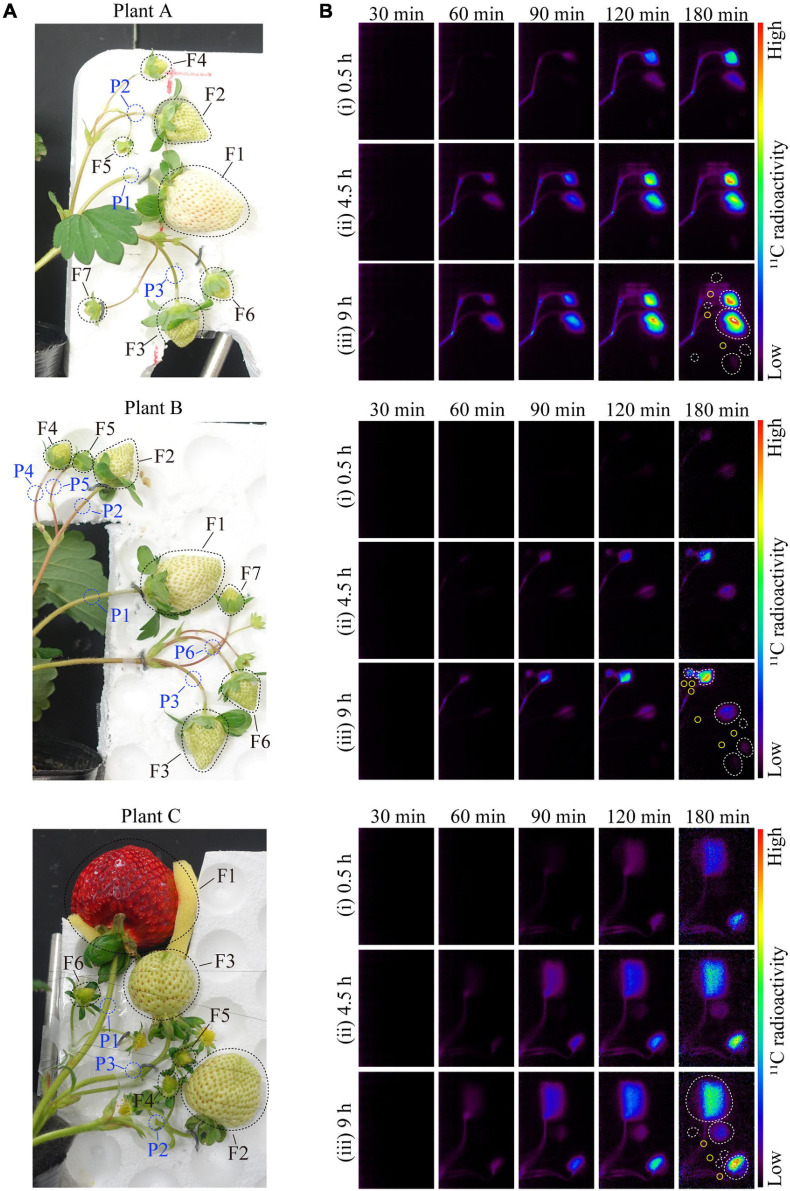
**(A)** Image of strawberry fruits from Plants A–C in the PETIS field of view. The black- and blue-dotted ellipses indicate the regions of interest (ROIs) on the fruits and pedicels, respectively. The F and P numbers in the figure indicate the fruit and pedicel numbers, respectively. The positions of the fruits on the same inflorescence are as follows: Fruit 1, primary; Fruits 2 and 3, secondary; and Fruits 4–7, tertiary. **(B)** Several PETIS images of ^11^C-photosynthate translocation into the fruits of Plant A for the (i) 0.5-h, (ii) 4.5-h, and (iii) 9-h lighting treatments. Each image represents the 5-min average for the time period indicated at the top of each PETIS image, which indicates the time elapsed since the start of PETIS imaging. The white and yellow ellipses indicate the ROIs of the fruits and pedicels, respectively.

June-bearing strawberry (*Fragaria* × *ananassa* Duch. cv. Fukuoka S6) plants were grown in a 37-m long × 9-m-wide plot in a greenhouse (37 m long × 27 m wide × 4.5 m high) at the NARO Kyushu Okinawa Agricultural Research Center, Japan (33°18.4′N, 130°32.8′E). Nursery plants were selected from the mother plant in early June and transplanted into plastic pots (6 cm diameter and 0.2 L volume). Connections to the mother stocks were retained through runners. Pots were filled with a substrate consisting of peat moss, coconut shells, and charcoal [3:5:2 (v/v/v)] and placed on a nursery bench. The plants were irrigated with water until rooting when their runners were cut from the mother stocks (late June). Thereafter, a nutrient solution (OK-F-1; OAT Agrio Co., Ltd., Tokyo, Japan; electrical conductivity = 0.6 dS m^–1^) was supplied at a rate of 300 mL d^–1^ plant^–1^. Nutrient supplementation was suspended from mid-August to mid-September to induce anthesis. During this time, plants were only supplied water. Flower buds had differentiated on the first inflorescences by mid-September, after which the plants were transplanted into substrate-filled plastic pots (0.15 m diameter and 2.6 L volume) and set on cultivation beds (30 m long × 30 cm wide × 80 cm high), with 20 cm between plants and 15 cm between rows. They were subsequently supplied with nutrient solution. The substrates and nutrient solutions were the same as those described above. The initial greenhouse ventilation temperature was 27°C. The air temperature was maintained above 8°C with a fuel-burning heater (HK2027TEV; NEPON Inc., Tokyo, Japan). Flowers were pollinated by bees. The plants with fruits were moved to a plant growth chamber at the Takasaki Advanced Radiation Research Center, National Institutes for Quantum and Radiological Science and Technology (QST), Japan (36°18.1′N, 139°04.4′E), where a PETIS was set up. The plants were cultivated for 2 weeks under the following experimental conditions: 12-h photoperiod, 400 μmol m^–2^ s^–1^ photosynthetically active radiation (PAR) using LEDs (ISL-150 × 150-HWW; CCS Inc., Kyoto, Japan), 20°C air temperature, 60% relative humidity, 380 μmol mol^–1^ CO_2_ concentration, and 300 mL d^–1^ plant^–1^ nutrient supply. After plants were acclimated to the experimental conditions, they were analyzed using the PETIS. In this study, three plants (Plants A–C) having similar growth conditions were used to ensure the high reproducibility of our translocation data. To simplify the relationship between the source leaves and sink fruits, we used test plants having primary inflorescences. Each plant had ten leaves. Plants A and B each had one primary fruit, two secondary fruits, and four tertiary fruits with a cymose inflorescence structure. Plant C had the same number of primary and secondary fruits as Plants A and B, but only had three tertiary fruits. Among all the fruits, the dry weights of the primary fruits (Fruit 1) were the greatest, followed by the secondary (Fruits 2 and 3) and tertiary fruits (Fruits 4–7), suggesting that the balance of fruit sizes in each plant was similar (Figure 1). Each fruit was divided into three different developmental stages: green, the first stage during which fruits are just fully formed; white, the second stage during which fruits begin to expand rapidly and become much larger; and red, the third stage during which fruit enlargement slows and the fruits become fully red. In Plants A and B, the primary fruits were in the white stage, the secondary fruits were just turning from the green stage to the white stage, and the tertiary fruits were in the green stage. In Plant C, the primary fruit was in the red stage, the secondary fruits were in the white stage, and the tertiary fruits were in the green stage (Figure 2A).

### ^11^CO_2_ Tracer Production

The ^11^CO_2_ tracer was produced by the ^14^N(p,α)^11^C reaction induced by bombarding pure nitrogen gas with 10 MeV protons from an AVF cyclotron located at Takasaki Ion Accelerators for Advanced Radiation Application (TIARA), QST, Japan (see Ishioka et al., 1999 for specific details). The irradiated gas containing nitrogen gas and ^11^CO_2_ was passed through a stainless steel trap (^11^CO_2_ trap) immersed in liquid nitrogen, and only the ^11^CO_2_ gas was collected as dry ice in the trap. In this study, approximately 160 MBq ^11^CO_2_ was collected and transferred to the gas circulation system for the PETIS imaging experiments.

### Imaging Experiments and Light Duration Treatments in the PETIS

The PETIS was installed in a plant growth chamber so that the ambient environmental conditions could be completely controlled during the experiments. Strawberry plants were positioned so that their fruits were located in the PETIS field of view (FOV) that was 119.9 mm wide and 187.0 mm high. The fruits were oriented correctly in the focal plane and positioned so they did not interfere with the mutual FOV. A fourth leaf developing immediately below the inflorescence was inserted into a gas-tied transparent acrylic box (exposure cell: 20 cm long, 15 cm wide, and 1 cm deep). The inlet of the exposure cell was connected to a ^11^CO_2_ trap, and approximately 160 MBq of ^11^CO_2_ was pumped into the cell at a constant rate of 100 mL min^–1^. The ^11^CO_2_ passed through the exposure cell within 1 min, and the remaining ^11^CO_2_ that was not fixed to the leaf was collected using soda lime (Wako Pure Chemical Industries Ltd., Osaka, Japan) in an acrylic tube connected to the outlet of the exposure cell. PETIS was started as soon as the ^11^CO_2_ was pumped out from the ^11^CO_2_ trap. At 20 min after the injection of ^11^CO_2_, the exposure cell was disconnected from the ^11^CO_2_ trap, and then, air in the growth chamber was pumped into the cell at a constant rate of 500 mL min^–1^ to continue the PETIS experiment. Thereafter, the ^11^C radioactivity collected in soda lime was measured using a Curie meter. The amount of assimilated ^11^C in the source leaf was calculated using the difference from the radioactivity of the injected ^11^CO_2_. PETIS images were acquired every 10 s for 180 min. The image data were automatically calibrated for the ^11^C decay assuming a half-life of 20.39 min and recorded on a personal computer (Figure 2B).

**FIGURE 3:**
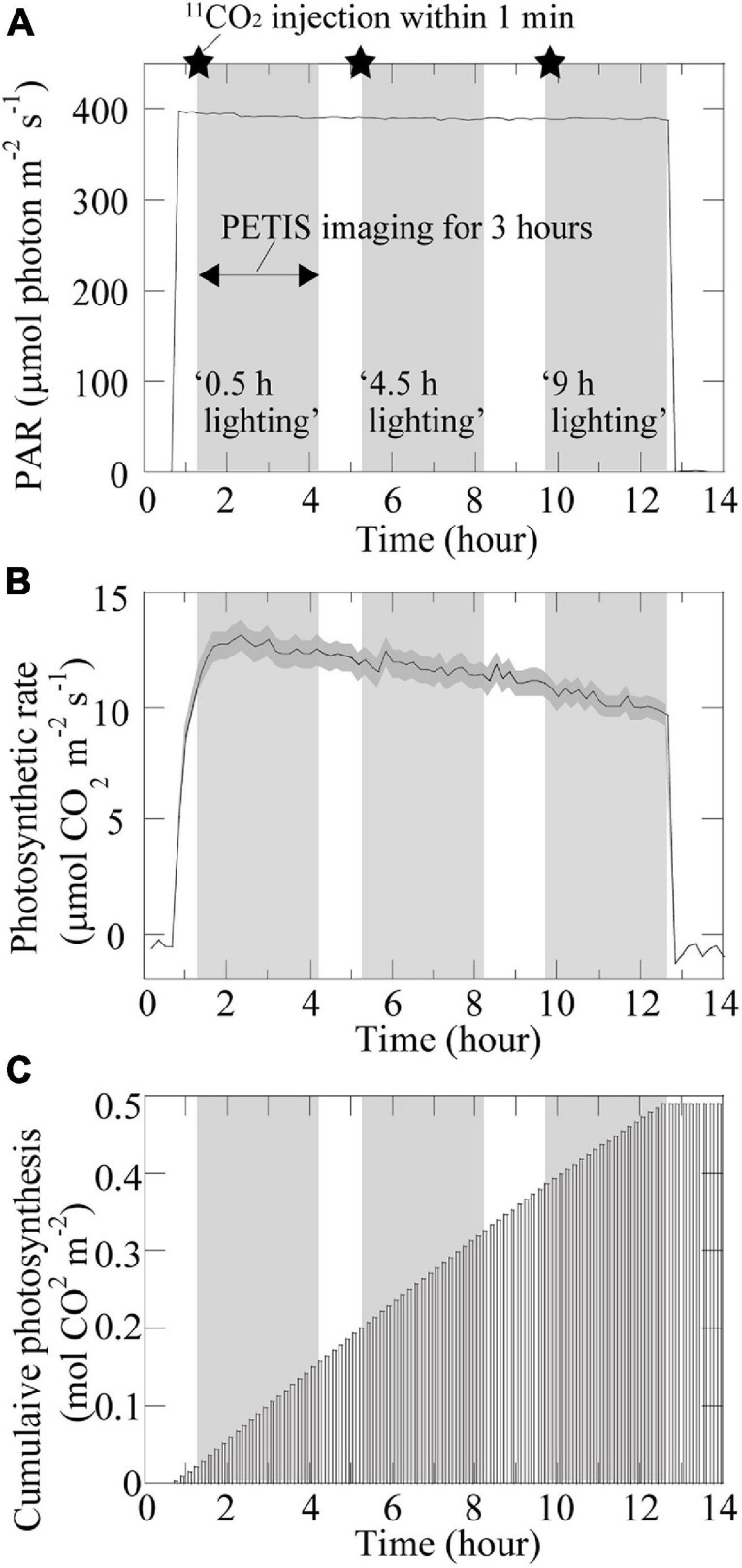
Photosynthesis-related time changes. Time changes in **(A)** photosynthetically active radiation (PAR). The setup of the lighting treatments is also shown. The star symbol indicates the timing of the ^11^CO_2_ injection; **(B)** photosynthetic rate; and **(C)** accumulated photosynthesis in the source leaf under specific environmental conditions during PETIS imaging. The dark gray area in **(B)** represents the standard deviation.

The 3-h PETIS imaging on the same plant was repeated three times during the 12-h light period. More specifically, at 0.5 h after changing the PAR on the adaxial surface of the source leaf from 0 to 400 μmol m^–2^ s^–1^, ^11^CO_2_ was fed to the source leaf within 1 min and the first 3-h PETIS imaging treatment was started (Figure 3A). The first PETIS treatment was named “0.5-h lighting.” At 1 h after the end of the first PETIS imaging treatment (i.e., at 4.5 h after changing to the light period), the second PETIS imaging treatment was started (“4.5-h lighting”) without changing the environmental conditions or the plant material. At 1.5 h after the end of the second PETIS imaging treatment (i.e., at 9 h after changing to the light period), the third PETIS imaging treatment was started (“9-h lighting”). The environmental conditions during the PETIS imaging were the same as the pre-experimental growth conditions. During the PETIS imaging experiments, the photosynthetic rates of the source leaves were determined every 10 min for 14 h (i.e., from 1 h before to 1 h after the 12-h light period) (Figures 3B,C). A portable photosynthesis system (model LI-6400; Li-Cor Inc., Lincoln, Nebraska, United States) was used under the following leaf chamber conditions: 20°C air temperature, 60% relative humidity, and 380 μmol mol^–1^ CO_2_ concentration. The PAR inside and outside the chamber was the same (400 μmol m^–2^ s^–1^) because of the natural light window at the chamber head. The air flow rate was maintained at 200 μmol s^–1^. At the beginning of the light period, the photosynthetic rate of the source leaves increased rapidly to approximately 13 μmol CO_2_ m^–2^ s^–1^ and then decreased slowly at a constant rate during the light period, reaching approximately 10 μmol CO_2_ m^–2^ s^–1^ by the end of the light period. The accumulated photosynthesis value increased almost linearly during the light period, reaching approximately 0.5 mol CO_2_ m^–2^ by the end.

### Translocation Analysis

**FIGURE 4:**
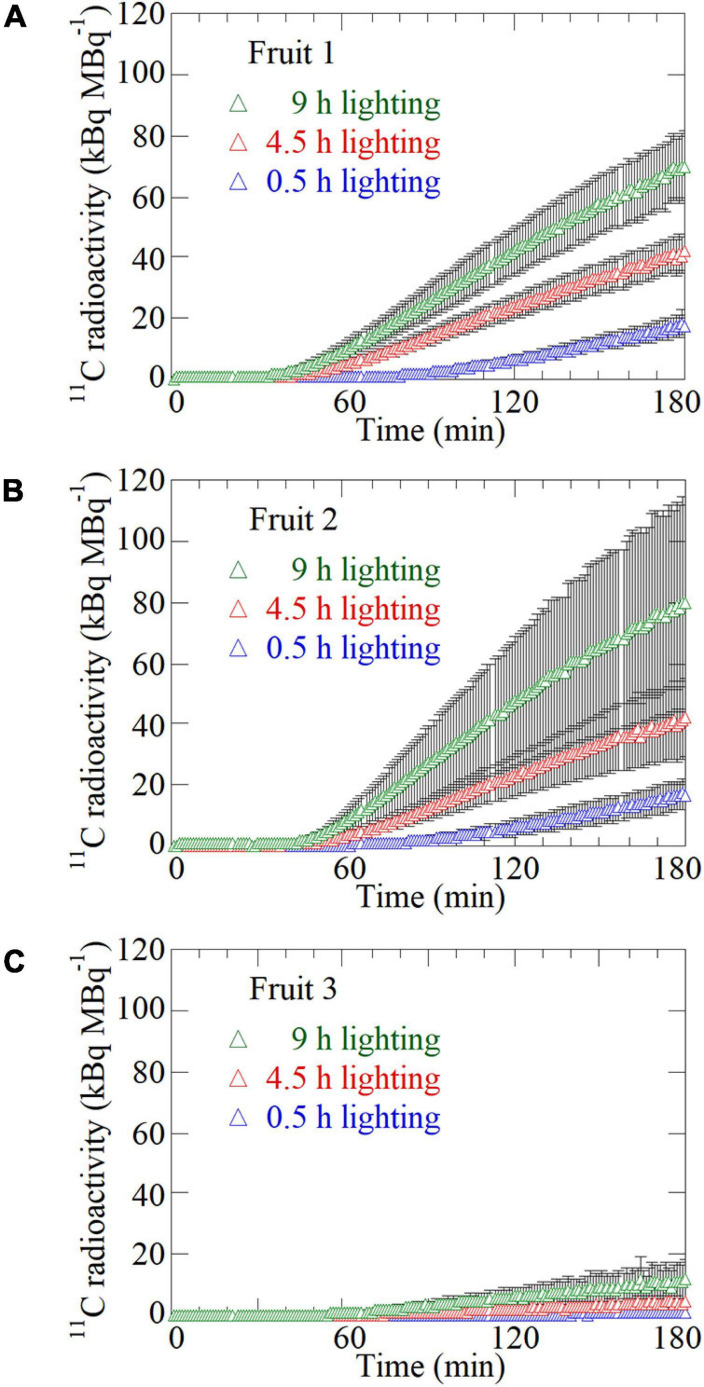
Time-course analysis of average ^11^C radioactivity in the ROIs of **(A)** Fruit 1, **(B)** Fruit 2, and **(C)** Fruit 3 for all the plants in response to the 0.5-, 4.5-, and 9-h lighting treatments. The black bar on each plot represents the standard deviation. The data were normalized against the ^11^C-radioactivity assimilated by the source leaf during each PETIS imaging experiment.

**FIGURE 5:**
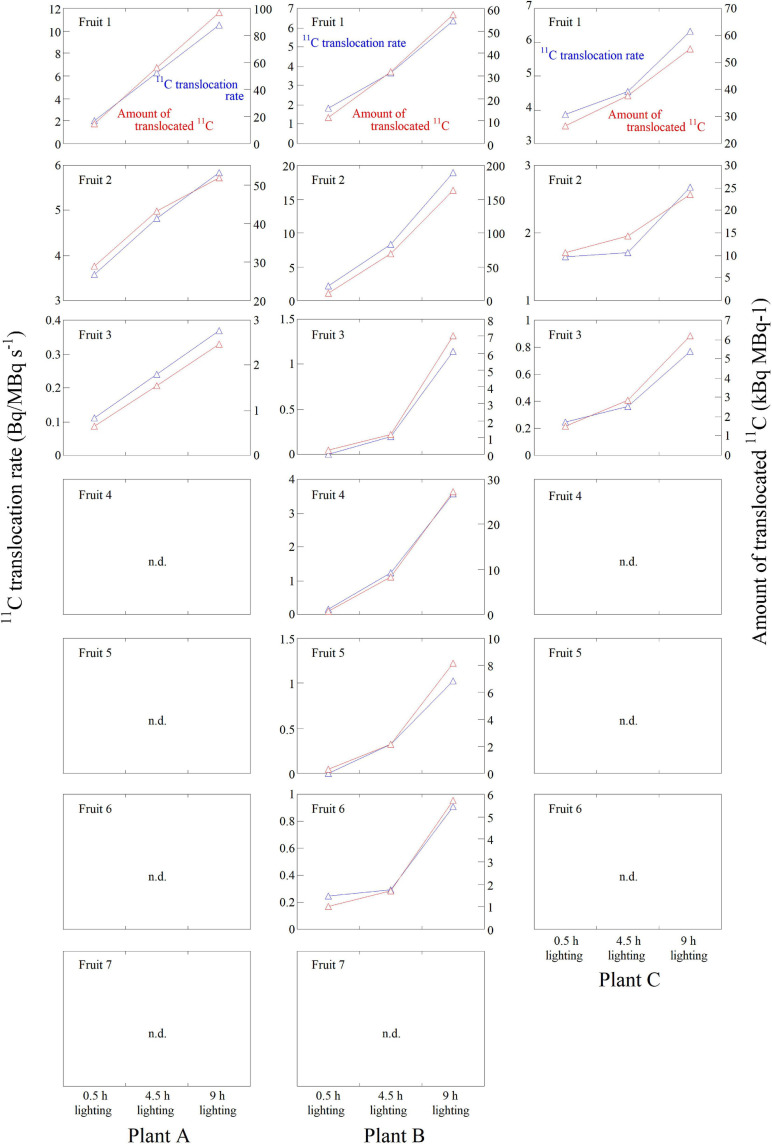
^11^C-photosynthate translocation rate and the amount of ^11^C-photosynthates translocated into all fruits in Plants A–C in response to the 0.5-, 4.5-, and 9-h lighting treatments. The data were normalized against the ^11^C-radioactivity assimilated by the source leaf during each PETIS imaging experiment. The fruits in which ^11^C-photosynthates were not detected are indicated with n.d.

**FIGURE 6:**
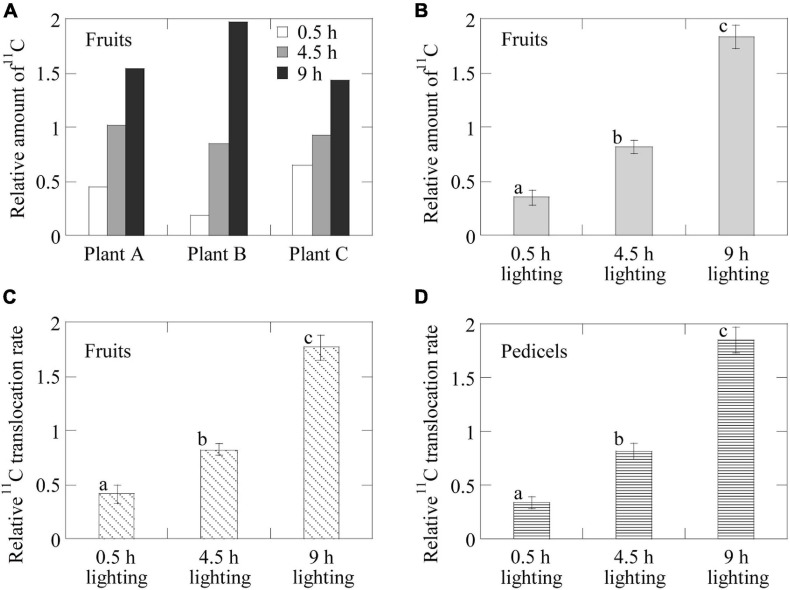
Average relative ^11^C-photosynthate translocation rates and the amounts of ^11^C-photosynthates translocated into the fruits having confirmed ^11^C-photosynthate translocation. Average relative amount of ^11^C-photosynthates translocated into the fruits **(A)** of each individual plant in response to the 0.5-, 4.5-, and 9-h lighting treatments and **(B)** in all the plants for each lighting treatment. Average relative ^11^C-photosynthate translocation rate **(C)** for the fruits in all the plants for each lighting treatment and **(D)** in the phloem of the pedicels of all the plants in response to the 0.5-, 4.5-, and 9-h lighting treatments. Different letters indicate significant differences (*p* < 0.05) as determined by the Tukey–Kramer test.

The ^11^C translocation into fruits was analyzed by setting the regions of interest (ROIs) around the fruits (white-dotted ellipses) and pedicel parts (yellow ellipses) in the PETIS images as shown in Figure 2B. Time-course analyses of ^11^C radioactivity (kBq) within each ROI were completed by generating time activity curves (TACs) from the signal intensities (counts per second) obtained using Image J (version 1.50) (National Institutes of Health, Bethesda, MD, United States)^1^ (Figure 4). The counting efficiency of the system (cps Bq^–1^) was then calculated. In this study, the ^11^C radioactivity in each fruit at the end of a PETIS imaging experiment reflected the amount of ^11^C-photosynthates translocated into each fruit (red line in Figure 5). Because photosynthates flow into, and accumulate in, fruits, the tracer increase rate (Figure 4) after the translocation of ^11^C into each fruit represents the accumulation rate of ^11^C in the fruit and, thus, the influx rate of photosynthates into the fruit. Therefore, we defined the increasing rate of ^11^C radioactivity in the fruits as the translocation rate of ^11^C-photosynthates into fruits (Figure 5). Furthermore, the translocation rate of ^11^C-photosynthate in the phloem was also calculated using the pedicel TAC data. These data were normalized against the ^11^C-radioactivity assimilated by the source leaf because of the differences between experiments regarding the amount of ^11^C taken up by the source leaf *via* photosynthesis (in kBq MBq^–1^). In addition, to confirm that individual differences in the ^11^C translocation rate and amount of translocated ^11^C to sink fruits were large, the changes after light duration treatments were comparatively analyzed. Specifically, the normalized amount of translocated ^11^C and normalized ^11^C translocation rate for each light duration treatment were divided by the corresponding average of the three light duration treatments. The average value of the relative amount of translocated ^11^C for fruits per plant was determined and compared with values from the light duration treatments (Figure 6A). Similarly, the relative amounts of translocated ^11^C and relative ^11^C translocation rates of all the fruits (Figures 6B,C) and all the pedicels (Figure 6D) in which ^11^C translocation was confirmed were averaged over all the plants in each light duration treatment.

**FIGURE 7:**
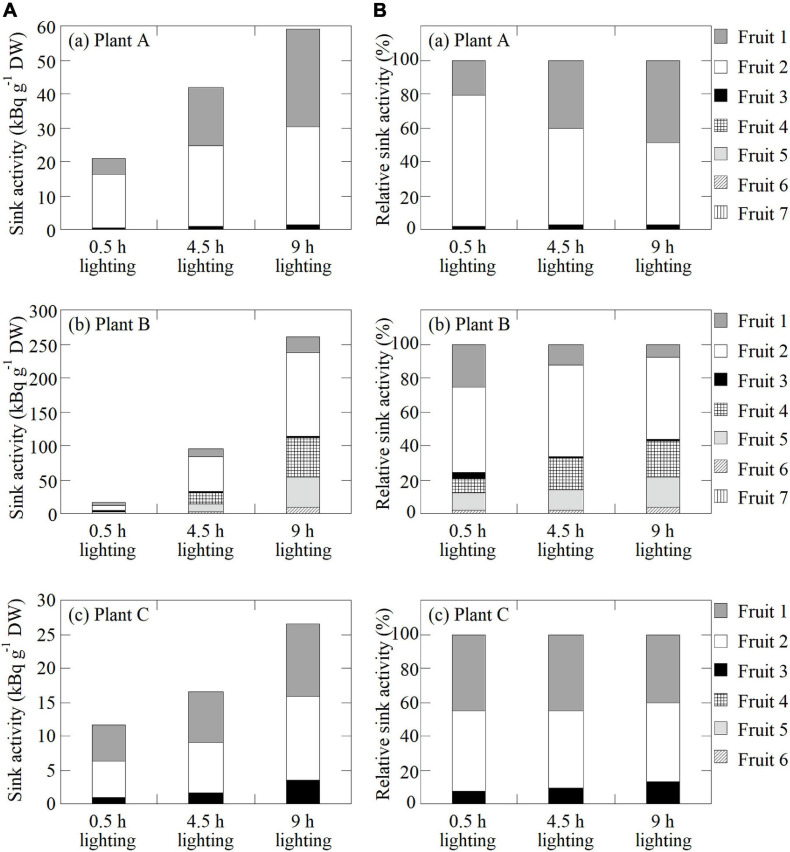
**(A)** Sink activity of all fruits in (a) Plant A, (b) Plant B, and (c) Plant C in response to the 0.5-, 4.5-, and 9-h lighting treatments. **(B)** Relative sink activity of all fruits in (a) Plant A, (b) Plant B, and (c) Plant C for each lighting treatment.

The sink activity of each fruit in each plant was evaluated for each treatment by normalizing the total amount of ^11^C radioactivity detected in each fruit against its dry weight as described in the study by Hidaka et al. (2019) (Figure 7A). Furthermore, the relative sink activity was calculated by dividing the sink activity of each fruit by the total sink activity of all fruits on an inflorescence in each plant (Figure 7B).

### Statistical Analysis

The relative ^11^C translocation rate and the relative amount of ^11^C translocated into fruits were calculated on the basis of the ^11^C radioactivity in the fruits. The ^11^C-photosynthate translocation was confirmed in three replicate plants (Plants A–C) (*n* = 12). Furthermore, the relative ^11^C translocation rate in the phloem was calculated using the pedicels of the fruits (*n* = 12). After the arcsine transformation of the original data, the significance of the differences between means (*p* < 0.05) among the different light duration treatments was determined by a Tukey–Kramer test using the transformed data. Statistical analyses were performed using the “Rcmdr” package (version 2.6-2) of the R software (R 3.6.3)^2^.

## Results

### Dynamic Analysis of ^11^C-Photosnthate Translocation

In Figure 2, an image (Figure 2A) shows strawberry fruits in the PETIS FOV and the averages of several PETIS images taken over 5 min (Figure 2B). The ^11^C-photosynthate translocation into strawberry fruits through pedicels was successfully visualized by PETIS imaging. It was visually confirmed that ^11^C-photosynthates translocated first to Fruit 2 and then to Fruit 1 in all the plants. The ^11^C-photosynthate translocation to fruits in the bunches was not homogeneous, and the translocation to Fruits 1–3 in Plants A and C, and to Fruits 1–6 in Plant B, were visually confirmed after the 9-h lighting treatment. The translocation of ^11^C-photosynthates to other fruits was not confirmed. Additionally, ^11^C-photosynthates did not accumulate uniformly in fruits. More specifically, the PETIS images revealed that they accumulated mainly in the upper sides of Fruit 1 from Plants A and B, and in Fruit 2 from Plant C, but also in the lower sides of Fruit 2 from Plants A and B. Additionally, they accumulated in the right sides of Fruit 3 from Plant B and of Fruit 1 from Plant A. There were no observable differences in the translocation patterns into fruits among the light duration treatments. In all the fruits in which ^11^C-photosynthate translocation was confirmed, the radioactivity of ^11^C at 180 min was the highest after the 9-h lighting treatment, followed by the 4.5- and 0.5-h lighting treatments.

Figure 4 presents the average TACs for all the plants after ^11^CO_2_ feeding in each ROI of Fruit 1 (Figure 4A), Fruit 2 (Figure 4B), and Fruit 3 (Figure 4C) in which ^11^C-photosynthate translocation was confirmed. The data from all the TACs were normalized against the ^11^C assimilation in the source leaf. For all the fruits, ^11^C-translocation was most prominent after the 9-h lighting treatment, followed by the 4.5-h and then the 0.5-h lighting treatments. In Fruit 1, the ^11^C tracer began to increase at approximately 53, 28, and 24 min after ^11^CO_2_ feeding in the 0.5-, 4.5-, and 9-h lighting treatments, respectively. In Fruit 2, the ^11^C tracer began to increase at approximately 49, 33, and 28 min after ^11^CO_2_ feeding in the 0.5-, 4.5-, and 9-h lighting treatments, respectively. In Fruit 3, the ^11^C tracer began to increase at approximately 68, 47, and 43 min after ^11^CO_2_ feeding in the 0.5-, 4.5-, and 9-h lighting treatments, respectively.

Figure 5 presents the ^11^C-photosynthate translocation rate and the amount of translocated ^11^C-photosynthates for each lighting treatment for all fruits of Plants A–C. In Plant A, ^11^C-photosynthate translocation was observed in three fruits. In Plants B and C, ^11^C-photosynthate translocation was detected in six and three fruits, respectively. In these fruits, both the ^11^C-photosynthate translocation rate and the amount of translocated ^11^C-photosynthates were the highest for the 9-h lighting treatment and the lowest for the 0.5-h lighting treatment.

Figure 6A presents the average relative amounts of ^11^C-photosynthates translocated into fruits per plant for each lighting treatment. Because of the large variation in the ^11^C-photosynthates translocation dynamics among individuals (Figure 4), we analyzed the relative values, i.e., the rates of change owing to the light duration treatments. In all plants, the relative amount of translocated ^11^C-photosynthates was the highest for the 9-h lighting treatment and the lowest for the 0.5-h lighting treatment. In Plant A, the average values for the 4.5- and 9-h lighting treatments were about 1.4 and 2.2 times higher than the corresponding values for the 0.5-h lighting treatment, respectively. In Plant B, the average values for the 4.5- and 9-h lighting treatments were about 4.6 and 10.8 times higher than those for the 0.5-h lighting treatment, respectively, whereas in Plant C, the average values for the 4.5- and 9-h lighting treatments were about 2.3 and 3.4 times higher than those for the 0.5-h lighting treatment, respectively. Figure 6B presents the average relative amounts of ^11^C-photosynthates translocated into fruits for all plants. The relative amounts of translocated ^11^C-photosynthates (±SE) were 0.35 ± 0.07, 0.82 ± 0.06, and 1.83 ± 0.11 for the 0.5-, 4.5-, and 9-h lighting treatments, respectively. The differences among treatments were significant. Figure 6C presents the average relative rates of ^11^C-photosynthates into fruits for all plants. The relative translocation rates (±SE) were 0.41 ± 0.08, 0.82 ± 0.05, and 1.76 ± 0.12 for the 0.5-, 4.5-, and 9-h lighting treatments, respectively. The differences among treatments were significant. The average relative ^11^C translocation rates in the pedicels of all plants also significantly increased with increasing daylight integrals (Figure 6D) and were 0.34 ± 0.06, 0.81 ± 0.07, and 1.85 ± 0.12 (±SE) for the 0.5-, 4.5-, and 9-h lighting treatments, respectively.

### Sink Activity

Figure 7A presents the sink activity of each fruit on each plant for all lighting treatment. In all fruits, the sink activity was at the highest and lowest for the 9- and 0.5-h lighting treatments, respectively. In all plants, the sink activity of Fruit 2, which is the secondary fruit, was at the highest. Figure 7B presents the relative sink activity of each fruit of each plant for all lighting treatments. In Plant A, the relative sink activity of Fruit 2 decreased with increasing daylight integrals, whereas the relative sink activity of Fruits 1 and 3 increased. In Plant B, the relative sink activity of Fruits 1–3 decreased with increasing daylight integrals, in contrast to the increasing relative sink activity of Fruits 4–6. In Plant C, the relative sink activity of Fruits 1 and 2 decreased slightly with increasing light exposure time, whereas the relative sink activity of Fruit 3 increased slightly.

## Discussion

During protected cultivation, photosynthate translocation dynamics should be considered when establishing the environmental control system to optimize yield and quality. Accordingly, the daily changes in photosynthate translocation dynamics must be determined. In this study, we successfully visualized the spatiotemporal variability in ^11^C-photosynthates translocation into strawberry fruits in response to increasing daylight integrals using the non-invasive analysis of ^11^C tracer and the PETIS, and also visualized the images of ^11^C tracer movement into the same strawberry fruits repeatedly during all the treatments of light period (Figure 2B). Furthermore, by analyzing the obtained PETIS images, the photosynthate translocation dynamics from one source leaf to an individual fruit could be evaluated separately with high spatiotemporal resolution (Figures 4, 5). Previous studies that assessed daily changes in photosynthate translocation monitored the movement of the ^14^C tracer fed to leaves at a specific time point during the light period at long time intervals. Pearson (1974), for example, estimated the net carbon dioxide exchange and loss of ^14^C-photosynthates from *Vicia faba* L. leaves throughout the day *via* infrared gas analysis and Geiger–Muller tube monitoring, respectively. The results of this earlier study indicated that, of the total carbon fixed in a 24-h period, about 50% is transferred in the current photoperiod, 14% in the dark period, and 5% in the next photoperiod. Sharkey and Pate (1976) investigated the diurnal changes in the leaf sugar concentration and the amount of ^14^C in the phloem sap from the distal tips of fruits attached to *Lupinus albus* L. plants by supplying ^14^CO_2_ to the leaves. In their study, the leaf sugar concentration increased, reaching peak levels in the afternoon, and the sugar output rate from the cut phloem of a fruit was directly related to the current leaf sugar content. Shishido et al. (1987, 1989) investigated the diurnal variation in the carbon budget of each organ in cucumber and tomato plants using a ^14^C tracer. They reported that the translocation started early in the morning and was much greater in the light period than in the dark period. Lanoue et al. (2018) also analyzed the effect of the intensity and quality of the light received by tomato leaves on the diurnal variation in photosynthate translocation using a ^14^C tracer. Their data indicated that photosynthate translocation in response to a lighting treatment involving all wavelengths, including orange and green wavelengths, was greater than that at night. Furthermore, the translocation under light varied from 65 to 83% of the total daily carbon fixed by leaf photosynthesis, depending on the light intensity. Most of these previous studies have analyzed the differences between light and dark periods to determine the diurnal variation in photosynthate translocation, and they proved that the translocation is greater under light than in darkness. Although long-term translocation dynamics have been analyzed, few studies have been analyzed at the short-time scale under the continuous lighting, which is an accelerating condition of photosynthesis, on the leaf surface with high spatial resolution. To the best of our knowledge, this study is the first to analyze the spatiotemporal variability of photosynthate translocation from one source leaf to each individual fruit during the light period with high spatiotemporal resolution.

The images obtained from the PETIS indicated that ^11^C-photosynthates were translocated from source leaves to several fruits, but not homogeneously. Furthermore, the imaging analysis revealed that ^11^C-photosynthates from CO_2_-fed leaves entered the fruits earlier as the light period increased (Figure 4). Hidaka et al. (2019) reported that ^11^C-photosynthates from source leaves reach fruits 52 min after ^11^CO_2_ feeding in strawberry plants. Although the plants used in this study were of the same size and cultivar as those used by Hidaka et al. (2019), the translocation into fruits occurred much earlier in our study depending on the duration of the light period (e.g., 4.5- and 9-h lighting treatments). In most of fruits in which ^11^C-photosynthates translocation was confirmed, both the photosynthate translocation rate and the amount of translocated photosynthates increased as the duration of the light treatment increased (Figure 5), and a significant difference was confirmed in the average value of the increasing rate (Figure 6). This is considered to be because of an increase in the concentration of sucrose, which is a translocated sugar in strawberries, in the source leaves during the light period. Photosynthate translocation between source and sink organs is generally considered to be driven by an osmotically generated pressure gradient, which is a mechanism known as Münch’s flow (Münch, 1930; Nobel, 2009). Considering the effect of increases in the strawberry leaf sucrose concentration on photosynthate translocation in terms of Münch’s flow, an increase in the leaf sucrose concentration leads to an increase in the frequency of encounters between sucrose and sucrose transporters on the plasma membrane as well as an increase in the amount of sucrose loaded into vascular bundles *via* active transport (Hammond and White, 2008). This decreases the osmotic water potential of the phloem and increases the water flow from the xylem to the phloem, thereby increasing the turgor pressure of the phloem (Lalonde et al., 2003, Schepper et al., 2013). Finally, a high-pressure gradient is generated and the phloem contents are pushed toward the sink organs (Maynard and Lucas, 1982; Lalonde et al., 1999; Hölttä et al., 2006), resulting in an enhanced photosynthate translocation from the sources to the sinks. Miyoshi et al. (2017) analyzed the same strawberry cultivar used in this study and determined that increases in the intensity of the light received by the source leaves increase concentration of the leaf sucrose and promotes the photosynthate translocation from the leaves. Under the continuous lighting conditions of the PETIS imaging experiment, the photosynthetic rate was relatively stable during the light period, and the cumulated photosynthesis in the source leaves increased almost linearly (Figures 3B,C). This could suggest that the leaf sucrose concentration increased with increase in the duration of the light period, resulting in promotion of the photosynthate translocation from the source leaf. The average relative rate of ^11^C-photosynthates translocation through the pedicels increased as the light period increased (Figure 6D), indicating that the loading rate from the source leaves increased during the light period. This is considered to be a compelling evidence to support the hypothesis of this study that the promotion of translocation to the fruit with the increasing daylight integrals on leaf surface is explained by an increase in the concentration of sugar in the leaf.

The sink activity of each fruit increased along with the light period (Figure 7A). We hypothesize that this is because the photosynthate-loading ability of the source leaf increased along with the daylight integrals, thereby promoting their translocation into each fruit. The sink activity level varied among fruits, with Fruit 2 (i.e., secondary fruit) having the highest sink activity among all the plants and treatments. Hidaka et al. (2019) analyzed the photosynthates translocated from a leaf just below the fruit bunch to fruits and observed that a secondary fruit had the highest sink activity, which is consistent with our results. Hidaka et al. (2019) speculated that the factor causing sink activities to differ among fruits is the strength of the auxin signals that are emitted from fruits and are critical for strawberry fruit growth (Nitsch, 1950). These signals vary depending on the developmental stage and the position of the sink fruit. Bangerth (1989) hypothesized that the exportation of polar auxin from earlier fruits on the same inflorescence inhibits its export from the later fruits. However, the highly reproducible findings of this study showed that when the developmental stage of the first fruit (the earlier fruit) in strawberry plants was red or white and each secondary fruit was white, or just turning from green to white, the sink activity of the secondary fruit (the later fruit) was stronger than that of the first fruit. This indicates that auxin export and inhibitory mechanisms are not uniform and vary depending on the developmental stage of each fruit, supporting the hypothesis described in the study by Hidaka et al. (2019). The balance of the relative sink activity levels among fruits also varied during the light period (Figure 7B). Fruit 2 in all the plants having higher relative sink activities in response to the 0.5-h lighting treatment tended to have the same or lower relative sink activities throughout the light period, whereas fruits with lower relative sink activities in response to the 0.5-h lighting treatment (Fruits 1 and 3 of Plant A, Fruits 4–6 of Plant B, and Fruit 3 of Plant C) tended to have higher relative sink activities that increased along with the light period duration. This implies that photosynthates are preferentially translocated to fruits having higher sink activities at the beginning of the light period and that the translocation into fruits with lower sink activities is accelerated during the latter part of the light period. Sink activity is subtly regulated in response to the cumulative photosynthate translocation to each fruit, and it has been suggested that auxin export from fruits and its inhibitory mechanisms vary not only with the fruit developmental stage but also within a short time span during the light period. Actually, in fruits having higher sink activity levels (Fruit 2 of Plant A, Fruits 1 and 2 of Plant B, and Fruit 1 of Plant C), the photosynthate translocation rates increased almost linearly or the increase rates under the 4.5- to 9-h lighting treatments were slightly lower than those under the 0.5- to 4.5-h lighting treatments, whereas in fruits having lower sink activity levels (Fruits 4–6 of Plant B and Fruit 3 of Plant C), the increase rates from the 4.5- to 9-h lighting treatments were higher than those from the 0.5- to 4.5-h lighting treatments (Figure 5). This supports the hypothesis that the light time period when photosynthate translocation occurs varied among fruits. The ability to study such subtle dynamic changes in sink activity is an advantage of PETIS and ^11^C tracer experiments.

A comparison of the relative sink activities among fruits on each plant for each lighting treatment (Figure 7B) indicated that the order of sink activities among fruits did not change during the light period. For fruits in which photosynthate translocation was not observed in response to the 0.5-h lighting treatment, photosynthate translocation remained undetectable even as the light period duration increased. The cause of the non-uniform distribution pattern of the photosynthate translocation to fruits over time may be the individual vascular system’s link between the source leaf and each fruit. Shishido et al. (1988) and Kikuchi et al. (2008) proposed in tomato and eggplant, respectively, that photosynthate translocation into sink fruits is affected by the vascular system’s link between source and sink organs. Hidaka et al. (2019) also supported the hypothesis that similar individual vascular connections exist between organs in strawberry plants. Furthermore, here, it was confirmed that the photosynthate translocation rate increased along with daylight integrals at source leaf surfaces in all the fruits in which the translocation was confirmed (Figure 5). Thus, the effects of physiological functional changes in the source leaves and the effects of promoting translocation from a source leaf were exerted in the same way on all the fruits. This suggests that there is an individual direct route between a source leaf and each sink fruit, representing the vascular system connection. In this study, we traced photosynthate translocation from the same source leaves, and photosynthate translocation to different fruits may be confirmed if the translocation from different source leaves is traced. Future studies are needed to prove this hypothesis. Comparisons between the ^11^C translocation rate and the amount of ^11^C translocated to individual fruits among plants revealed that their levels differed greatly between Plants A and B, even though the fruit positions and their developmental stages were similar. Moreover, in Plant B, the rate and amount of ^11^C-photosynthate translocation to the whole fruit bunch were greater than those of the other plants (Figure 5). We speculate that this resulted from the different developmental stages of the fruits on each plant. Yamazaki et al. (2015) reported that in tomato, the sink strength of the whole fruit bunch is greater when a number of fruits at younger stages are set in the bunch. In this study, all the fruits in Plant B were at earlier developmental stages than those of the other plants at the same positions (Figure 2A), and thus, the sink strength of the whole fruit bunch on Plant B was greater than those of the other plants, resulting in differences in ^11^C translocation levels. This hypothesis also explains why Fruit 1 of Plant B (Figure 7B) did not follow the trend of fruits having low sink activities during the 0.5-h lighting treatment showing high sink activities during the 4.5- and 9-h lighting treatments. The sink strength of the entire fruit bunch of Plant B was greater than those of the other plants, which may have led to an increased number of fruits in which ^11^C translocation was observed. As a result, the number of competing sink fruits in the fruit bunch increased in Plant B compared with the other plants, and it was assumed that ^11^C translocation to other young fruits was prioritized over the oldest fruit (Fruit 1).

## Conclusion

The spatiotemporal variability in the ^11^C-photosynthates translocation from individual source leaves into each strawberry fruit *in vivo* under increasing daylight integrals at leaf surface was visualized non-invasively and in real time using the PETIS. The obtained images revealed that photosynthate translocation from a source leaf differed among fruits at various positions of the same inflorescence. Moreover, the distribution pattern of photosynthates translocated into fruits did not vary during the light period. These results indicate that the correspondence between source leaves and sink fruits is unaffected by changes in the light environment, but is mediated by the individual vascular connections between a source leaf and each sink fruit. Additionally, the photosynthate translocation from source leaf to sink fruits was promoted in response to increasing daylight integrals. Furthermore, the order of the sink activity did not change when the daylight integral increased, although the balance of the sink activity among fruits changed. These results suggest that photosynthates are preferentially translocated into fruits with a high sink activity at the beginning of the light period, but the translocation into fruits with a low sink activity increases later in the light period. This study is the first to use an ^11^C-radioactive isotope and a PETIS to elucidate the variability in the real-time translocation of photosynthates into individual fruits on strawberry inflorescences in response to increasing daylight integrals.

## Data Availability Statement

The raw data supporting the conclusions of this article will be made available by the authors, without undue reservation.

## Author Contributions

YM and KH conceived and designed the experiments, analyzed the data, and wrote the manuscript. YM, KH, Y-GY, NS, KK, and NK performed the experiments. All authors finalized the manuscript.

## Conflict of Interest

The authors declare that the research was conducted in the absence of any commercial or financial relationships that could be construed as a potential conflict of interest.

## Footnotes

1 http://rsb.info.nih.gov/ij/

2 http://cran.r-project.org/
