# Supplementary material for: Non-invasive 11C-Imaging Revealed the Spatiotemporal Variability in the Translocation of Photosynthates Into Strawberry Fruits in Response to Increasing Daylight Integrals at Leaf Surface
Source: Front Plant Sci. 2021 Jul 14;12:688887. doi: 10.3389/fpls.2021.688887 (PMC8317645; doi:10.3389/fpls.2021.688887)
Supplement: Supplementary file 2 [file Presentation_2.PPTX]

## Slide 1
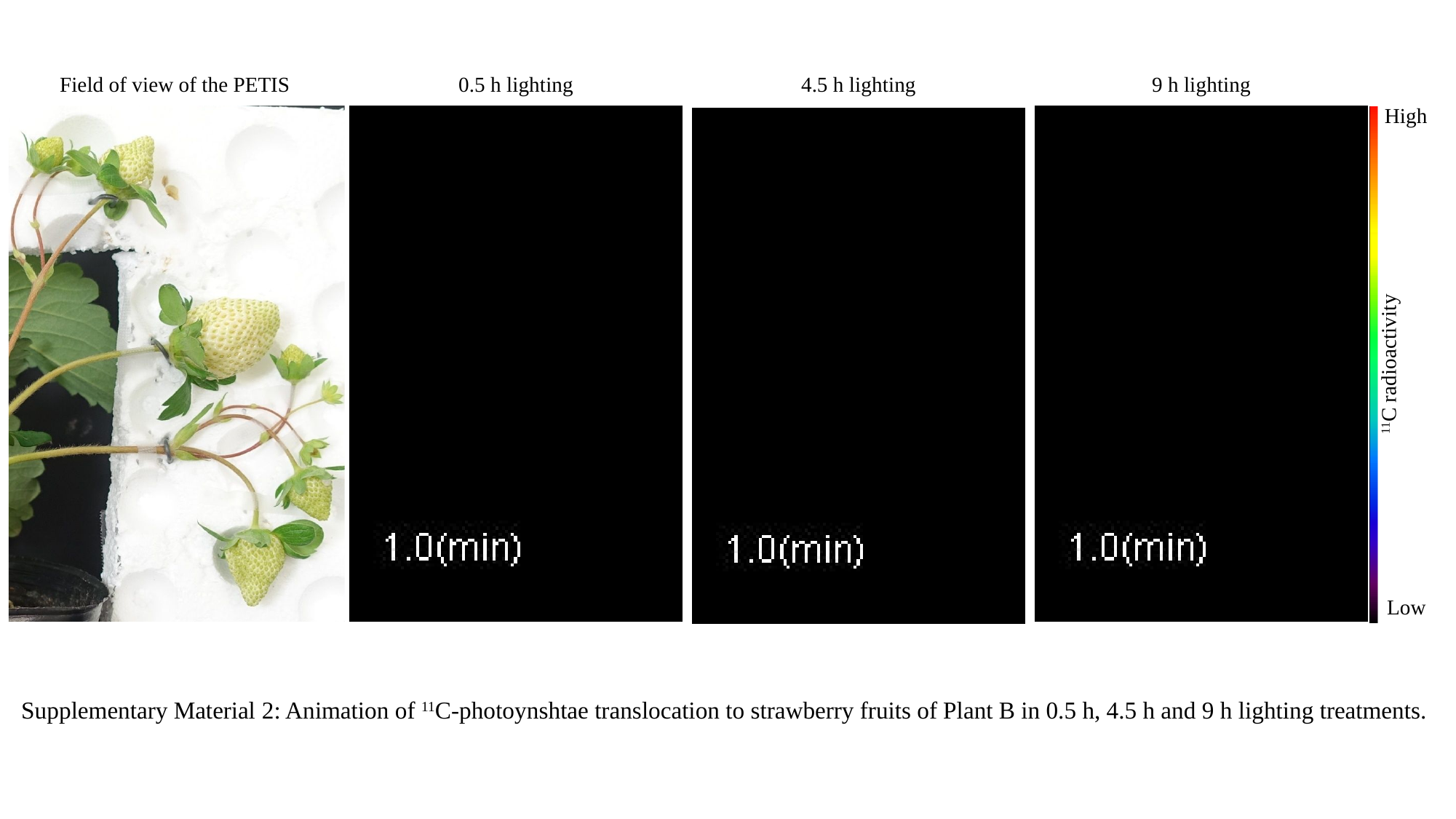

Field of view of the PETIS
0.5 h lighting
4.5 h lighting
9 h lighting
High
11C radioactivity
Low
Supplementary Material 2: Animation of 11C-photoynshtae translocation to strawberry fruits of Plant B in 0.5 h, 4.5 h and 9 h lighting treatments.
